# Neoadjuvant palbociclib on ER+ breast cancer (N007): clinical response and EndoPredict’s value

**DOI:** 10.1530/ERC-17-0396

**Published:** 2017-11-20

**Authors:** Louis W C Chow, Satoshi Morita, Christopher Y C Chow, Wai-Kuen Ng, Masakazu Toi

**Affiliations:** 1 State Key Laboratory of Quality Research in Chinese MedicinesMacau University of Science and Technology, Macau SAR, Macao; 2 Organisation for Oncology and Translational ResearchHong Kong SAR, China; 3 UNIMED Medical InstituteHong Kong SAR, China; 4 Department of Biomedical Statistics and BioinformaticsKyoto University Graduate School of Medicine, Kyoto, Japan; 5 Department of PathologyPrecious Blood Hospital, Hong Kong SAR, China; 6 Breast SurgeryKyoto University Graduate School of Medicine, Kyoto, Japan

**Keywords:** breast cancer, neoadjuvant, letrozole, palbociclib, EndoPredict

## Abstract

The purpose of the study was to test the efficacy of neoadjuvant palbociclib therapy and to evaluate its impact on cell cycle arrest and changes in EndoPredict (EP) scores before and after treatment. Postmenopausal women with histologically proven ER+ve, HER2−ve invasive breast cancer, 2 cm or greater, were enrolled in an open-label, single-arm study. Twenty eligible patients were given letrozole 2.5 mg per day together with palbociclib 125 mg per day for 3 out of 4 weeks in repeated cycles for 16 weeks (4 cycles) before surgery. The primary end points were clinical response rates (cRR) and preoperative endocrine prognostic index (PEPI). The secondary end points were pathologic response and gene expression testing with EP test on collected tumor samples. The following results were obtained. 17 patients showed a clinical response of 50% or more, including 8 complete responses and 9 partial responses. There was significant reduction in area (*P* < 0.0001) and volume (*P* = 0.017) of the cancer. Pathologic complete response (pCR) was achieved in one patient; all cancers were downgraded after treatment. Ki67 (*P* = 0.044) and EP scores (*P* < 0.0001) were significantly reduced after treatment. Analysis of the relative gene expression levels showed that all proliferative genes, IL6ST and RBBP8 were decreased after palbociclib treatment. 6 patients with intermediate and three patients with high PEPI risk scores were found to have low EPclin scores. All patients with high PEPI relapse risk score had high EPclin score. In conclusion, effective clinical response was demonstrated by neoadjuvant letrozole in combination with palbociclib. Compared with PEPI, EPclin might be a better parameter to estimate prognosis after neoadjuvant therapy.

## Introduction

The usefulness of endocrine therapy has been well demonstrated in the management of hormone-sensitive breast cancer (Ellis *et al.* 2011). The neoadjuvant ACOSOG Z1031 trial showed a high clinical response rate of 63–75% in postmenopausal women with clinical stage II or III estrogen receptor (ER)-positive breast cancer (Ellis *et al.* 2011). Nevertheless, endocrine resistance is still a concern for this group of patient; additional therapy might be a way out to improve clinical outcome.

Presence of CDK4-associated kinase activity was implicated in breast tumorigenesis (Yu *et al.* 2006). Direct analyses of primary tumors have revealed loss of Rb expression in 20–35% of tumors, and loss of heterozygosity or other alterations of the Rb locus in 7–37% of tumors (Borg *et al.* 1992, Pietiläinen *et al.* 1995, Oesterreich & Fuqua 1999, Chano *et al.* 2002). In preclinical models, Rb depletion appears to be associated with resistance to anti-estrogen therapy (Bosco *et al.* 2007). The loss of p16INK4a, low expression of CDK inhibitors p21 and p27, and high expression level of cyclin E and D1 have all been associated with resistance to anti-estrogen therapy (Musgrove 1995, Cariou *et al.* 2000, Zhou 2005, Nair 2008).

Palbociclib, a CDK-4/6 inhibitor, is an orally active potent and highly selective reversible inhibitor of CDK4 and CDK6, prevents cellular DNA synthesis by prohibiting the progression of the cell cycle from G1 into the S phase. The activity of CDK-4/6 inhibitor was demonstrated in luminal ER+ subtype cell lines (Finn *et al.* 2009).

The Paloma studies have shown that palbociclib combined with aromatase inhibitor or anti-estrogen resulted in longer progression-free survival than hormonal therapy alone among patients with hormone-receptor-positive metastatic breast cancer who had progression of disease during prior endocrine therapy (Finn *et al.* 2015, Turner *et al.* 2015). The treatment was generally well tolerated and the most common adverse events included neutropenia, leucopenia and fatigue (Slamon *et al.* 2010).

Pathologic complete response (pCR) is still a surrogate marker for prognosis in neoadjuvant studies. However, neoadjuvant endocrine therapy for 3–4 months only achieved less than 3% of pCR (Chow *et al.* 2008). Clinical response via measurable parameters during neoadjuvant endocrine therapy was used as an important primary endpoint. However, it is not clear whether this response correlates with relapse or breast cancer death risks. Ki67 proliferation index has been implicated as a surrogate marker of response for neoadjuvant hormonal therapy. A score of 2.7 or less was regarded as complete cell cycle arrest (Ma *et al.* 2015).

The preoperative endocrine prognostic index (PEPI) score was derived from tumor characteristics present after women with stage 2 and 3 breast cancer underwent four months of neoadjuvant hormonal therapy (P024 and IMPACT trials) (Ellis *et al.* 2008). It took into consideration four factors of the surgical samples after neoadjuvant therapy to estimate risk of relapse and survival rates. They included the size of the residual cancer, the nodal status, the Ki67 proliferation index and the estrogen receptor status (Allred score). The scoring process produced three groups (risk score 0, 1–3 and ≥4) that were associated with relapse-free survival of 10, 23 and 48% and breast cancer-specific survival of 2, 11 and 17%, respectively.

The EndoPredict (EP) test provides a multigene score that combines the expression levels of proliferative and ESR1 signaling/differentiation-associated genes. It provides additional prognostic information for the identification of early and late distant relapses beyond what can be achieved by combining the commonly used clinical parameters in ER-positive and HER2-negative breast cancer patients. The EPclin score could also identify a subgroup of patients who have an excellent long-term prognosis after 5 years of endocrine therapy (Dubsky *et al.* 2013).

The current study aims to test the efficacy of neoadjuvant hormonal treatment in combination with palbociclib. We also evaluate the impact of palbociclib on cell cycle arrest as well as changes in gene expression levels and EP scores before and after treatment. We compare the PEPI and EPclin scores on the post-treatment surgical specimens, as a predictive tool of prognosis after neoadjuvant hormonal therapy.

## Methods

### Patient selection

Postmenopausal women with histologically proven ER-positive, HER2-negative invasive breast cancer, 2 cm or greater in tumor size, were invited to join the study. They had to be of Eastern Cooperative Oncology Group (ECOG) Performance Status of 1 or below and be able to give written informed consent. The kidney and liver functions should be within normal limits and the left ventricular ejection fraction should be 60% or more. Male patients, patients allergic to the tested drugs, and patients on concomitant medication with strong inhibition or enhancement of CYP3A4 were excluded. Patients with locally advanced cancers or with distant metastasis were also excluded.

### Study design

This was an open-label, single-arm study to determine the efficacy and safety of neoadjuvant letrozole-CDK-4/6 inhibitor combinational regimen in ER-positive, HER2-negative breast cancer in postmenopausal women. Eligible patients were given letrozole 2.5 mg per day together with palbociclib 125 mg per day for 3 out of 4 weeks in repeated cycles for 16 weeks (4 cycles) before surgery. Palbociclib was stopped 1 week before surgery, whereas letrozole was continued till day of surgery. The primary end points of the study were clinical response rates (cRR) and PEPI. The secondary end points were pathologic response and gene expression testing with EP test on collected tumor samples at surgery.

The study, including the protocol, informed consent form and other information to subjects, was reviewed by a registered institutional review board.

### Efficacy and safety assessment

Baseline tumor assessment was carried out within 4 weeks before the start of treatment. This assessment included bilateral mammography, breast ultrasound and whole body positron emission tomography (PET) scan.

cRR were evaluated clinically by bi-dimensional clinical measurements and radiologically by ultrasound before the start of treatment and at week 16. Clinical response (complete response and partial response) was evaluated using World Health Organization/International Union Against Cancer (WHO/UICC) criteria and radiological response was evaluated using Response Evaluation Criteria In Solid Tumors (RECIST) guideline, version 1.1. The pathologic response and the pathology of the resected specimen at the time of surgery were evaluated and assessed by a well-qualified pathologist.

The PEPI score includes size of the residual cancer, number of involved lymph nodes, the value of Ki67 and Allred score at the time of surgery (Pietiläinen *et al.* 1995). Patients were classified into low, intermediate and high-risk groups.

The EP assay is based on the quantification of 8 cancer-related genes of interest (GOI: *BIRC5, UBE2C, DHCR7, RBBP8, IL6ST, AZGP1, MGP* and *STC2*) and 3 normalization genes (*CALM2, OAZ1* and *RPL37A*) in formalin-fixed and paraffin-embedded (FFPE) tissue sections by quantitative reverse transcription polymerase chain reaction (RT-PCR) (Filipits *et al.* 2011). The EP score was derived from relative expression levels of these genes. The combination of the EP score with two clinical risk factors, which are nodal status and tumor size, results in the EPclin score. EP and EPclin low-risk and high-risk categories were already pre-specified in the previous study (Turnbull *et al.* 2015). Patients with an EP score <5 (EPclin score <3.3) were classified as low risk for distance recurrence, whereas patients with an EP score ≥5 (EPclin score ≥3.3) were stratified as high risk.

Safety assessments consisted of monitoring and recording all adverse events and serious adverse events. The patients were monitored regularly with complete blood count and blood chemistry. Vital signs and performance status were also measured after each cycle of treatment.

All subjective toxicities encountered during the study were evaluated according to the National Cancer Institute-Common Terminology Criteria for Adverse Events, v3.0.

Clinical and laboratory data were collected at the central trial office. Differences in parameters were assessed using paired *t*-test. All statistical analyses were conducted using SPSS statistical software.

## Results

Twenty postmenopausal patients were enrolled into the study. The mean age of the patients was 63.3 years (age range 51–82 years). The cancers were all ductal and T2 on largest diameter. The patient demographics are tabulated in Table 1.

**Table 1:** Demographics of patients.

	**No. of patients**
Age (mean, range)	63.3 (51–82) years
Size (mean, range)	3.59 (2.89–4.89) cm
Grade (I vs II vs III)	11/6/3
ER+PR+ vs ER+PR−	17/3
Nodal status (positive vs negative)*	10/10

*Nodal status on PET scan.

### Clinical response rates

Seventeen (85%) patients showed a clinical tumor response of 50% or more, including 8 (40%) complete responses and 9 (45%) partial responses. Analysis of the area of the cancer on bi-dimensional measurement before and after treatment showed that the mean values were 11.61 cm^2^ and 2.91 cm^2^, respectively. The change was substantial and statistically significant (*P* < 0.0001).

**Figure 1:**
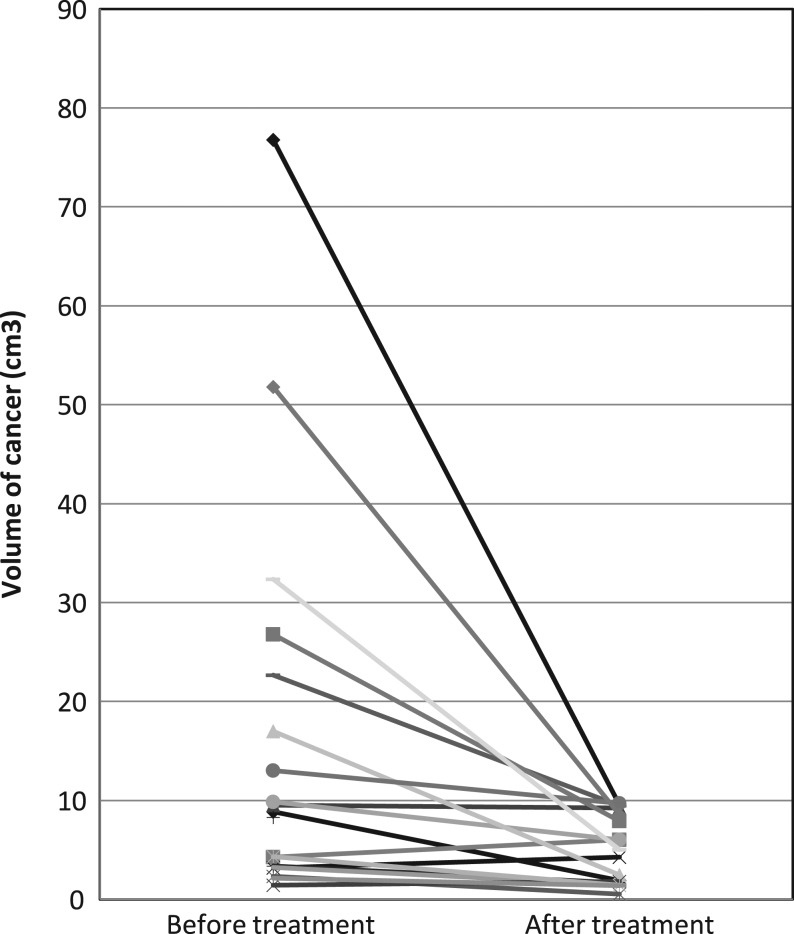
The volume of the cancer on three-dimensional measurement before and after treatment with neoadjuvant letrozole in combination with palbociclib. Each line represents the data for individual patient.

Fourteen patients (70%) showed response on ultrasound. The volume on three-dimensional measurement before and after treatment can be seen in Fig. 1. The mean values were 15.85 cm^3^ and 4.91 cm^3^ respectively. The difference was statistically significant (*P* = 0.017).

### Pathologic response

There were three invasive lobular cancers, one with mixed invasive ductal and lobular, and the remaining sixteen were all invasive ductal cancers. There were eleven grade 1, six grade 2 and three grade 3 cancers on core biopsies before treatment. The pathology of the surgically resected specimens showed 14 grade I cancers and 5 grade II cancers. Pathologic complete response (pCR) was achieved in one patient with only residual intermediate grade ductal carcinoma in situ (DCIS). All cancers were downgraded after treatment.

There were nine patients with positive axillary lymph nodes on PET scan before treatment. The nodal status remained the same after treatment, except for two patients who had fewer nodal metastases.

### Change in Ki67

**Figure 2:**
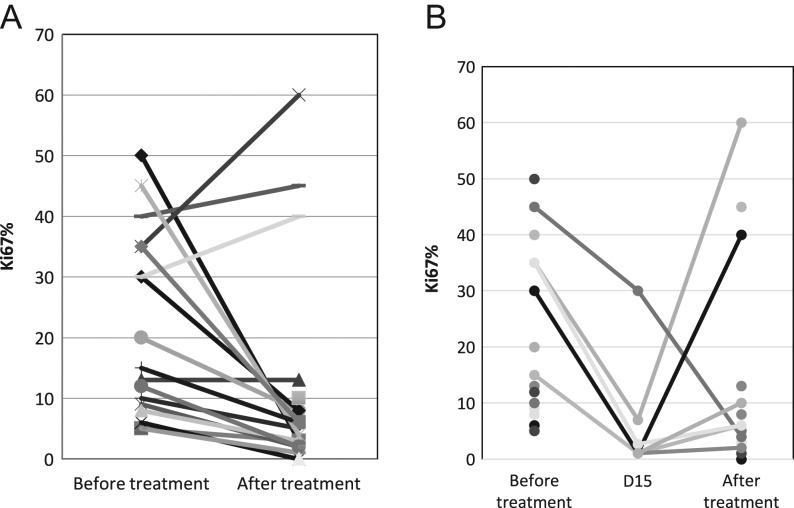
(A) Overall change in Ki67 before and after treatment with neoadjuvant letrozole in combination with palbociclib. Each line represents the data for individual patient. Ki67 >15% was considered as high proliferation fraction. (B) Change in Ki67 before treatment, on day 15 and after treatment with neoadjuvant letrozole in combination with palbociclib. Each line represents the data for individual patient. Ki67 >15% was considered as high proliferation fraction.

The change in Ki67 before and after treatment is shown in Fig. 2A. The mean value of Ki67 before treatment was 21.65%; after treatment it was 11.35% (*P* = 0.044). Core biopsy was performed on seven patients on day 15 of cycle 1 for the evaluation of Ki67. It was less than 2.7 in five of seven (71.4%) patients (Fig. 2B). However, only eight of 20 (40%) patients had a Ki67 less than 2.7 at the time of surgery after treatment. Except for three patients with elevation after treatment, the other 17 patients showed a significant drop in value. Eight of 17 (47.1%) patients had a high proliferation fraction (Ki67 >15%) before treatment but only three of 20 (15%) patients were determined to have Ki67 over 15% after treatment.

### Change in relative expression levels (dCt) of gene of interest

**Figure 3:**
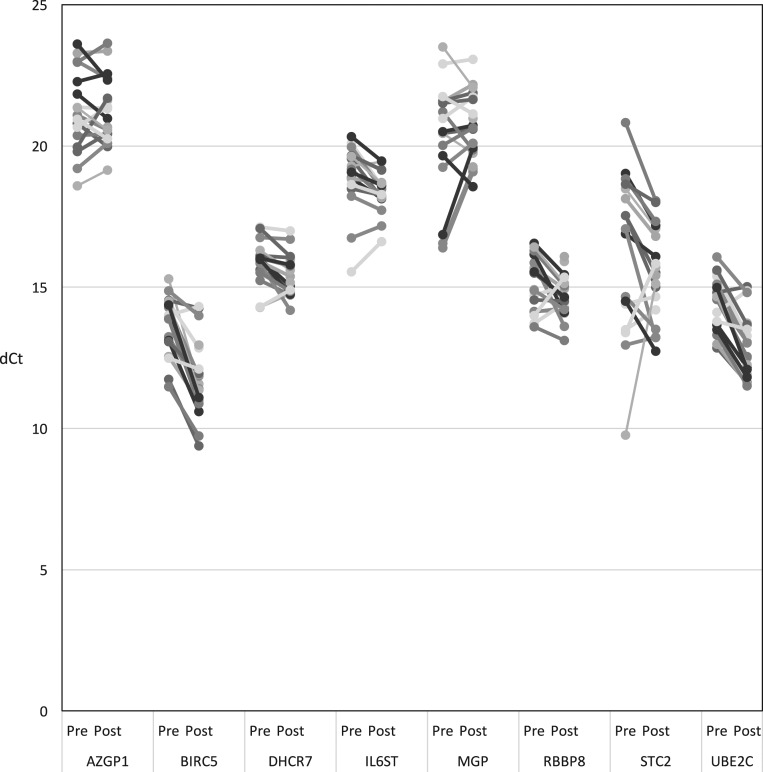
Change in relative gene expression level (dCt) of gene of interest. Each level represents the data for each individual patient.

The change of dCt of each of the eight cancer-related genes before and after treatment were shown in Fig. 3. The three proliferation-associated genes (*BIRC5, UBE2C* and *DHCR7*) showed statistically significant reduction in levels (*P* < 0.001 for all three) after palbociclib treatment.

Of the other five hormone receptor-associated genes, there were significant decrease in levels of IL6ST (*P* = 0.0034) and RBBP8 (*P* = 0.021).

### Change in EP score

**Figure 4:**
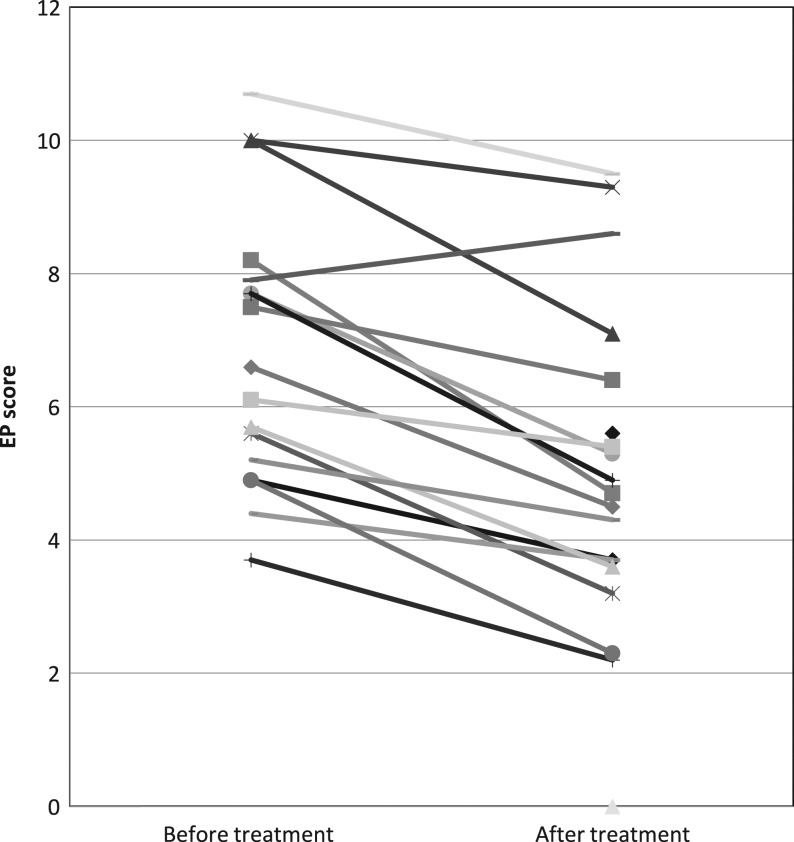
Change in EP score before and after treatment with neoadjuvant letrozole in combination with palbociclib. Each line represents the data for individual patient. EP score <5 was classified as low risk for distance recurrence; EP score ≥5 was stratified as high risk. EP, EndoPredict.

Fourteen of 17 (82.35%) patients had a high EP score of 5 or above before treatment, but only nine out of 19 (47.37%) had a high EP score after treatment (Fig. 4). Three patients with low EP before treatment continued to have low EP after treatment. Seven more patients showed a low EP after treatment. The mean EP before treatment was 6.87; after treatment it was 5.25 (*P* < 0.0001).

### PEPI score

**Figure 5:**
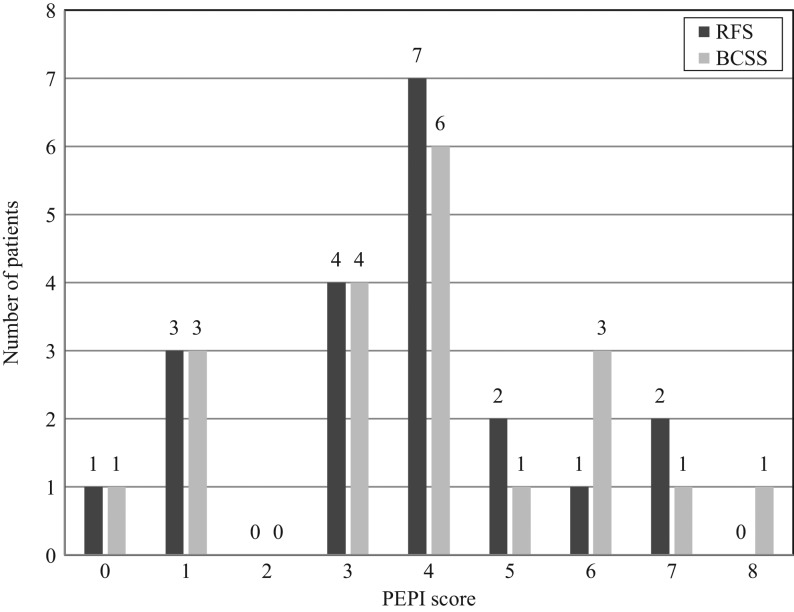
Distribution of PEPI scores. A PEPI score of 0 predicts a low risk of recurrence; a score of 1–3 predicts an intermediate risk of recurrence; a score of 4 or higher predicts a high risk of recurrence. BCSS, breast cancer-specific survival; PEPI, preoperative endocrine prognostic index; RFS, relapse-free survival.

The distribution of the PEPI scores is shown in Fig. 5. There was only one patient with score 0, seven with a score between 1 and 3 and twelve with a score 4 or more, for both relapse-free survival (RFS) and breast cancer-specific survival (BCSS).

### EPclin score

**Figure 6:**
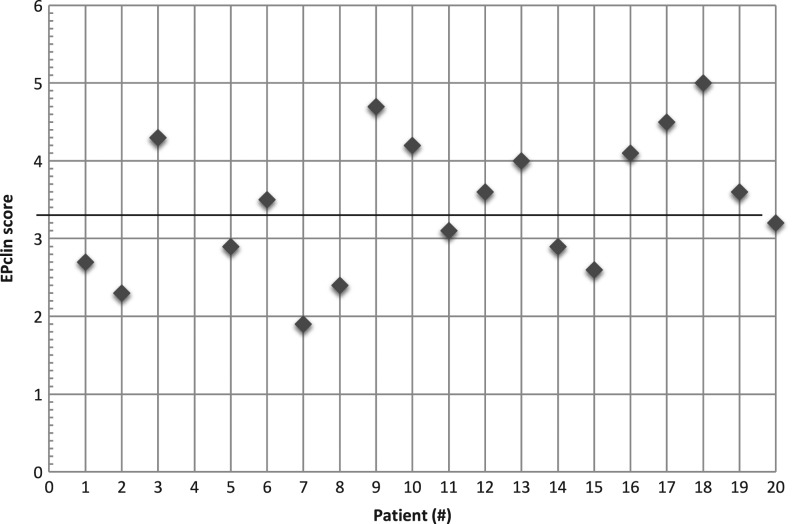
Distribution of EPclin scores. EPclin score <3.3 was classified as low risk for distance recurrence; EPclin score ≥3.3 was stratified as high risk. EPclin, EndoPredict-clin.

The distribution of the EPclin scores is shown in Fig. 6. One patient developed pCR and should be regarded as low risk. Nine of the other 19 (47.4%) patients had a score of less than 3.3 and thus were regarded as low risk. Patients with lobular cancers had high EPclin scores.

### Concordance between PEPI and EPclin scores

The results were tabulated in Table 2. There was no patient with low PEPI relapse risk score. Six patients with intermediate and three patients with high PEPI risk scores were found to have low EPclin scores. All other patients with high PEPI relapse risk score had high EPclin score.

**Table 2:** Concordance between PEPI and EPclin scores.

**PEPI**	**Low**	**Intermediate**	**High**	**Total**
EPclin				
Low	0	6	3	9
High	0	0	10	10
Total	0	6	13	19

### Adverse events

**Figure 7:**
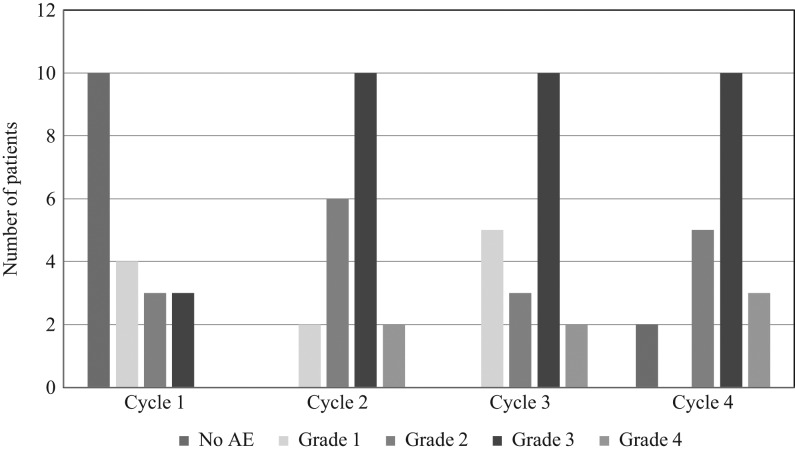
Neutropenia during the course of treatment. AE, adverse events.

The common adverse events were listed in Table 3. All patients developed afebrile neutropenia during the course of treatment (Fig. 7). Most patients developed neutropenia after the first cycle. More than half of the patients developed persistent grade 3 or 4 neutropenia, requiring adjustment of dose or suspension of therapy. Other adverse events include bodily pain/discomfort, mucositis/stomatitis, skin rash/itchiness and hand-foot syndrome. These were mostly grade 1 or 2.

**Table 3:** Common adverse events during the course of treatment.

	**Grade 1** (%)	**Grade 2** (%)	**Grade 3** (%)	**Grade 4** (%)
Neutropenia	14 (70)	18 (90)	16 (80)	6 (30)
Thrombocytopenia	2 (10)	1 (5)	–	–
Hand-foot syndrome	3 (15)	–	–	–
Skin rash and itchiness	4 (20)	2 (10)	–	–
Bodily pain/discomfort	8 (40)	–	–	–
Mucositis/stomatitis	7 (35)	–	–	–
Abnormal liver function	–	2 (10)	2 (10)	–
Fatigue	2 (10)	–	–	–
Insomnia	2 (10)	–	–	–

## Discussion

Palbociclib is an oral CDK4/6 inhibitor, useful in the management of ER-positive and HER2-negative metastatic breast cancer when combined with an aromatase inhibitor or fulvestrant. Our study showed that the combination treatment may also be active in the neoadjuvant setting. The clinical response rate was 85%, with 40% being complete response. The ultrasound response rate was 70%, which was higher than the expected of 50%. All cancers were downgraded on histological evaluation and one patient developed pCR, which is not common after neoadjuvant hormonal therapy.

In a recent neoadjuvant trial combining palbociclib and anastrozole, the cRR was 67%, with slightly lower complete response (Ma *et al.* 2015). Its primary endpoint was complete cell cycle arrest, defined as Ki67 2.7%, on C1D15 biopsy. This biologic endpoint was chosen because increasing levels of Ki67 on neoadjuvant therapy were previously found to be associated with an increased risk of relapse in the long term. 87% of the patient population on palbociclib achieved complete cell cycle arrest; however, Ki67 levels rebound after 4 weeks of washout before surgery.

In our study, similar findings were observed. In seven patients who consented to C1D15 biopsy, five showed complete cell cycle arrest. However, this was only observed in 40% of patients at the time of surgery, after a short period of washout, suggesting a rebound in Ki67 levels. Nonetheless, the post-treatment Ki67 values were statistically lower than the pre-treatment levels.

In an attempt to search for a surrogate biomarker of prognosis after neoadjuvant hormonal therapy, the tissue samples were tested with EP test, which analyzed the disease-free survival based on the expression levels of proliferative and ESR1 signaling/differentiation-associated cancer genes (Dubsky *et al.* 2013). EP test was based on genetic analysis (RT-PCR) instead of immunohistochemical (IHC) analysis of the protein expression of Ki67. This will reduce the intrinsic limitation of IHC staining. The measurement of expression levels of three proliferation genes (*BIRC5, UBE2C* and *DHCR7*), makes EP a more reliable and objective test than the sole measurement of Ki67 protein with IHC. EP test also provides the alternate analysis of cell cycle arrest and reduce the impact of Ki67 rebound during the washout period. The test was performed on core biopsy and surgical samples.

We found that the EP scores were reduced significantly after treatment. This was related particularly to the reduction in levels of the proliferative genes, IL6ST and RBB8P. While it is expected that the treatment will have an impact on proliferation, the change in IL6ST suggests that the effects of the combination may also be associated with immune signaling. IL6ST has been implicated to play important role in cytokine–receptor interaction, an enriched pathway related to lympho-vascular invasion (Klahan *et al.* 2017). It is also one of four other genes identified to be predictive of response to aromatase inhibitor therapy (Turnbull *et al.* 2015). RBBP8 gene encodes the retinoblastoma-binding protein 8. It is found among several proteins that bind directly to retinoblastoma protein (RB1), which regulates G1/S transition of cell proliferation (Fusco *et al*. 1988). RB1 is also influenced by palbociclib via inhibition of CDK4/6. Thus, measurement of *RBBP8* and EP could be useful surrogate of response for the compound. This will require a larger clinical trial and further translational research for validation.

PEPI score might be a useful prediction tool of survival after neoadjuvant hormonal therapy; however, this may not be applicable when patients were treated with palbociclib in addition to hormonal therapy, since there is usually a washout period for the drug before surgery. Ki67 levels usually rebound, and this would affect the analysis of cell cycle arrest and survival. In our study, none of the non-pCR patients was found to have a low risk with the score of 0 and an associated relapse risk of less than 10%.

EPclin incorporates EP genetic score as well as other clinical parameters, i.e. residual cancer size and nodal status: two parameters that are also included in the PEPI score. Unlike Ki67, the genetic score is unlikely to rebound during the washout period and thus, should be a better parameter to estimate the survival after neoadjuvant therapy. There were 9 patients with EPclin low risk, with an associated relapse rate of 10% or less in 10 years.

Concordance analysis showed that PEPI high-risk category correlated well with EPclin high-risk group. But the other nine patients with PEPI intermediate or high risk were actually EPclin low risk. As the test differs mainly on the analysis of proliferation fraction, the rebound in Ki67 during the washout might be the key reason of the discrepancy. Further study with larger sample size and longer follow-up period should be conducted to evaluate whether EPclin can replace PEPI as the surrogate marker of prognosis after neoadjuvant hormonal therapy.

The good response is not associated with serious adverse events, except for afebrile neutropenia, which was present during the course of four cycles of treatment. Grade 3 or 4 neutropenia is not uncommon, requiring dose reduction or interruption. Interestingly, this occurs more often after the first cycle and persists till the fourth cycle.

## Declaration of interest

The authors declare that there is no conflict of interest that could be perceived as prejudicing the impartiality of the research reported.

## Funding

Pfizer Inc. provided financial support for this study, including the supply of palbociclib. Pfizer Inc. is not the study sponsor and not responsible for study design, data collection and analysis, decision to publish or preparation of the manuscript.

## Author contribution statement

Louis W C Chow: Design and implementation of the clinical trial, preparation and writing of manuscript. Satoshi Morita: Advice on statistical analysis. Christopher Y C Chow: Preparation of references. Wai-Kuen Ng: Pathologic evaluation of specimens. Masakazu Toi: Design of the clinical trial.
